# GenomeTrakr proficiency testing for foodborne pathogen surveillance: an exercise from 2015

**DOI:** 10.1099/mgen.0.000185

**Published:** 2018-06-15

**Authors:** Ruth E. Timme, Hugh Rand, Maria Sanchez Leon, Maria Hoffmann, Errol Strain, Marc Allard, Dwayne Roberson, Joseph D. Baugher

**Affiliations:** Center for Food Safety and Applied Nutrition, US Food and Drug Administration, College Park, MD, USA

**Keywords:** molecular epidemiology, proficiency test, whole-genome sequencing, outbreak detection, foodborne pathogen, GenomeTrakr

## Abstract

Pathogen monitoring is becoming more precise as sequencing technologies become more affordable and accessible worldwide. This transition is especially apparent in the field of food safety, which has demonstrated how whole-genome sequencing (WGS) can be used on a global scale to protect public health. GenomeTrakr coordinates the WGS performed by public-health agencies and other partners by providing a public database with real-time cluster analysis for foodborne pathogen surveillance. Because WGS is being used to support enforcement decisions, it is essential to have confidence in the quality of the data being used and the downstream data analyses that guide these decisions. Routine proficiency tests, such as the one described here, have an important role in ensuring the validity of both data and procedures. In 2015, the GenomeTrakr proficiency test distributed eight isolates of common foodborne pathogens to participating laboratories, who were required to follow a specific protocol for performing WGS. Resulting sequence data were evaluated for several metrics, including proper labelling, sequence quality and new single nucleotide polymorphisms (SNPs). Illumina MiSeq sequence data collected for the same set of strains across 21 different laboratories exhibited high reproducibility, while revealing a narrow range of technical and biological variance. The numbers of SNPs reported for sequencing runs of the same isolates across multiple laboratories support the robustness of our cluster analysis pipeline in that each individual isolate cultured and resequenced multiple times in multiple places are all easily identifiable as originating from the same source.

## Data Summary

Raw data collected as part of the proficiency testing exercise have been submitted to the National Center for Biotechnology Information (NCBI) Sequence Read Archive (SRA) under BioProject PRJNA386310 (https://www.ncbi.nlm.nih.gov/bioproject/386310). BioSamples and SRA run accession numbers for these data are listed in Table S1 (available in the online version of this article). Complete, annotated reference genomes were submitted to NCBI’s GenBank with the accession numbers listed in Table 2.

**Impact Statement**

Bacterial pathogen surveillance is now being supported by whole-genome sequencing (WGS) technology and, because WGS is being used to support enforcement decisions by public-health agencies, it is essential to have confidence in the quality of the data being used and the downstream data analyses that guide these decisions. Routine proficiency tests, such as the one described here, have an important role in ensuring the validity of both data and procedures. The exercise design and resulting data generated will be especially relevant to the global foodborne pathogen surveillance community, especially for non-USA countries looking to establish similar surveillance networks. It will also be relevant for researchers.

## Introduction

Foodborne pathogen surveillance in the USA is transitioning from pulsed-field gel electrophoresis (PFGE) to whole-genome sequencing (WGS) for subtyping and disease surveillance. The United States Food and Drug Administration (FDA), tasked with ensuring a safe food supply, began piloting this technology in 2012 with the formation of the GenomeTrakr network [1]. Microbial food and environmental isolates collected by the GenomeTrakr network laboratories (FDA field labs, state and local public-health labs, academic labs and others) are submitted to the public GenomeTrakr database hosted at the National Center for Biotechnology Information (NCBI). WGS data collection for foodborne pathogens is now part of the standard operating protocol (SOP) across the three federal agencies working in food safety [the FDA, the Centers for Disease Control and Prevention (CDC) and the United States Department of Agriculture – Food Safety and Inspection Service (USDA-FSIS)]. Unsurprisingly, single nucleotide polymorphisms (SNPs) gathered across the entire bacterial genome [2–4], as well as multi-locus sequence typing (cgMLST or wgMLST) [5, 6], provide far greater resolution for microbial pathogen surveillance than the current gold standard, PFGE. Genomic data collection and submissions to the NCBI Sequence Read Archive (SRA) are harmonized across all three federal agencies, ensuring data compatibility for various public-health activities (e.g. outbreak detection, outbreak monitoring, outbreak investigation, source tracking, drug resistance monitoring and evaluations of facility inspection results). Bacterial isolates such as *Salmonella enterica*, *Listeria monocytogenes, Escherichia coli, Vibrio parahaemolyticus* and *Campylobacter* spp. obtained from food and environmental sources are sequenced by GenomeTrakr laboratories in real-time. Once these data are in the SRA, genomes can be clustered into phylogenetic trees and results made publicly available at the NCBI’s Pathogen Detection website www.illumina.com/content/dam/illumina-marketing/documents/products/other/miseq-overclustering-primer-770-2014-038.pdf(https://www.ncbi.nlm.nih.gov/pathogens). As of April 13th 2018, approximately 210 000 genomes have been submitted from all over the world to this public pathogen surveillance network, including from the USA’s GenomeTrakr and PulseNet [6, 7] networks.

For each new technology, it must be ensured that data collection is accurate and reproducible − both within individual laboratories and across networks of laboratories − especially when those data are used in the service of public health [8–12]. WGS tools are being widely used for clinical testing purposes; applications of genome sequencing to human health require rigorous proficiency testing (PT) to ensure that the multiple laboratories performing diagnostic analyses do so consistently, and adhere to patient privacy requirements [13–15]. Multiple studies have also validated the application of genome sequencing to pathogen surveillance [2–4, 16–18] and further validation has been proposed via a new Clinical Laboratory Improvement Amendments (CLIA) protocol for performing Illumina WGS in public-health laboratories [19]. There are also significant efforts underway to establish and standardize global networks for real-time WGS sequencing and analysis to rapidly determine the relatedness of microbial isolates using a global database for these comparisons [20–22]. Real-time for this application means data are deposited into a public database immediately after sequencing, which contrasts with the traditional ‘hold until published’ model. As a first step, the Global Microbial Identifier (GMI) initiative held a series of PT exercises [20] comparing data collection and analysis in the following countries: the USA, the UK, Denmark, Canada, Germany, France, Malaysia, Italy, Sweden, Spain, Israel, Poland, Finland and Australia. That GMI PT dataset has recently been submitted to NCBI BioProject PRJEB21132. Despite the wide use of next-generation sequencing (NGS) in the public-health arena, the raw data collected as part of PT is rarely released publicly; thus, there’s a need for providing in-depth public analysis of PT NGS data collected across a distributed laboratory network.

The GenomeTrakr proficiency test (GTPT) was designed to assess the performance of participating laboratories and to help the FDA coordinating team identify areas for improvement (e.g. sequence quality, data transfer, following an SOP and communications). The third annual GTPT exercise was held in 2015, which included 26 participating laboratories (Table 1). Each laboratory received eight foodborne pathogen isolates (Table 2), selected to span both genomic diversity (multiple species) and complexity (containing zero to multiple plasmids). Each laboratory was responsible for performing their own standard culture preparation and DNA isolation, then for following the standard GenomeTrakr SOP for library preparation and sequencing. The raw sequence results from the PT exercise were submitted to the GenomeTrakr coordinating team, which used their internal PT analysis pipeline for a variety of quality control (QC) metrics; these metrics provided QC and assessed whether the WGS data returned by each laboratory were appropriate for use in downstream analytics. The results of the QC metrics and individual WGS data from each laboratory were compiled into reports that presented each laboratory's result metrics in relation to proficiency exhibited across the whole set of participating GenomeTrakr laboratories.

**Table 1.** Participating laboratories in the 2015 GTPT exercise

**Participating laboratories**
Arizona State Public Health Laboratory, TGen, USA
California Department of Public Health, USA
Centers for Disease Control and Prevention, Enteric Diseases Laboratory Branch, USA
FDA Arkansas Laboratory, USA
FDA Denver Laboratory, USA
FDA Northeast Food and Feed Laboratory, USA
FDA Pacific Northwest Laboratory, USA
FDA Pacific Southwest Laboratory, USA
FDA San Francisco Laboratory, USA
FDA Southeast Food and Feed Laboratory, USA
FDA Winchester Engineering Analytical Center, USA
FDA/CFSAN/Office of Applied Research and Safety Assessment, USA
FDA/CFSAN/Office of Regulatory Science, USA
Florida Department of Agriculture and Consumer Services, USA
Food Safety Laboratory, New Mexico State University, USA
Hawaii State Department of Public Health, USA
IEH Laboratories and Consulting Group, Lake Forest Park, WA, USA
Minnesota Department of Health, USA
Nestlé Research Center - Institute of Food Safety and Analytical Science, USA
New York State Department of Health – Wadsworth Center, USA
SENASICA – Servicio National De Sanidad, Inocuidad Y Calidad Agroalimentaria, Mexico
State of Alaska Public Health Laboratory, USA
Texas Department of State Health Service, USA
United States Department of Agriculture – Food Safety Inspection Service, USA
Virginia Division of Consolidated Laboratory Services, USA
Washington State Department of Health Public Health Laboratory, USA

**Table 2.** Summary information for the eight PT strains distributed as part of the exercise NCBI accession numbers are listed for both the reference genomes and the raw data collected from the PT exercise.

					NCBI accession no. for annotated complete reference genome			Raw data collected from PT exercise	
Organism	CFSAN ID	Genome size (bp)	No. of plasmids	G+C content (mol%)	BioSample	BioProject	Assembly	NCBI BioSample	NCBI BioProject
*Salmonella enterica* ser. Montevideo	CFSAN000255	4 694 375	0	53.4	SAMN00710608	PRJNA186035	ASM18895v5	SAMN07210937	PRJNA386310
*Salmonella enterica* ser. Heidelberg	CFSAN000318	4 951 478	3	53.4	SAMN01088008	PRJNA186035	ASM43010v1	SAMN07210938	PRJNA386310
*Listeria monocytogenes*	CFSAN001178	3 032 269	0	38.1	SAMN01816124	PRJNA215355	ASM19539v5	SAMN07210944	PRJNA386310
*Escherichia coli*	CFSAN002236	5 045 919	1	52.3	SAMN02147037	PRJNA230969	ASM46495v2	SAMN07210945	PRJNA386310
*Listeria monocytogenes*	CFSAN008100	3 108 121	1	38.1	SAMN02689388	PRJNA215355	ASM100592v1	SAMN07210931	PRJNA386310
*Shigella sonnei*	CFSAN030807	5 062 953	8	52.3	SAMN03612247	PRJNA273284	ASM244253v1	SAMN07210932	PRJNA386310
*Campylobacter coli*	CFSAN032805	1 750 173	2	31	SAMN03580886	PRJNA309864	ASM240714v1	SAMN07210933	PRJNA386310
*Campylobacter jejuni*	CFSAN032806	1 782 911	1	31	SAMN03580887	PRJNA309864	ASM240712v1	SAMN07210930	PRJNA386310

## Methods

### Participating laboratories and PT isolates

All GenomeTrakr laboratories and collaborators were invited to participate in the 2015 GTPT, although participation was voluntary. Of the 26 laboratories that participated in the 2015 GTPT, 21 correctly followed the GenomeTrakr SOP and were included in our analysis (Table 1). This group of 21 represented diverse laboratory types, including public-health laboratories (state, local, federal), academic laboratories, international laboratories and a few industry laboratories. The GTPT coordinating team distributed eight pure culture isolates as stabs in soft agar (trypticase soy agar; Becton) in September 2015 (Table 2). The isolates represented a wide taxonomic diversity, encompassing the most common foodborne pathogens submitted to GenomeTrakr: *Salmonella enterica* subsp. *enterica* serovar Montevideo, *Salmonella enterica* serovar Heidelberg*, L. monocytogenes* (two strains), *E. coli*, *Shigella sonnei*, *Campylobacter jejuni* and *Campylobacter coli*. The set of PT isolates provided opportunities for mislabelling, demanded a breadth of proficiency in culturing and DNA isolation, and allowed for a broad assessment of taxon-specific variability. Each laboratory was given 2 months to complete the sequencing exercise and transfer its data back to the GTPT coordinating team. These laboratories were instructed to run the 8 PT isolates in a single run with a total of 16 isolates. To achieve this, some laboratories ran the PT set twice (submitting both sets) and others filled the run with other non-PT microbial genomes. Laboratories that were not equipped to handle *Campylobacter* only sequenced the remaining six PT isolates.

### DNA extraction, library preparation and sequencing for PT data

Participating laboratories were instructed to use the standard GenomeTrakr SOP for library preparation and DNA sequencing, allowing for laboratory-specific variations in culturing and DNA isolation methods. The libraries were all prepared using the Nextera XT DNA library prep kit according to the manufacturer’s instructions and then sequenced for 2×250 cycles using the MiSeq platform (Illumina).

### Data transfer to FDA

There were three possible routes for transferring data back to the GTPT coordinating team. Laboratories with access to Illumina’s cloud service, BaseSpace, streamed their sequencing run(s) to BaseSpace, then made use of that service to share their data with the GTPT coordinating team, who could then download those data into the FDA computing environment. Non-FDA laboratories that did not have BaseSpace access transferred their raw data (fastq files) to the FDA through a secure cloud storage site. Laboratories within the FDA network transferred their run to a shared drive. All fastq files were assigned to their respective laboratory using automated workflows within a Laboratory Information Management System (LIMS), which provided data tracking and management.

### Generation of PT reference genomes

The following work was performed at the FDA to produce a complete, annotated, reference genome for each of the eight PT isolates. We first isolated the genomic DNA of each strain to be shared for the PT from overnight cultures using a DNeasy blood and tissue kit (Qiagen). These PT isolate genomes were closed using the Pacific Biosciences (PacBio) *RS II* sequencing platform, as previously reported [23]. Genomic DNA was sheared into approximately 20 kb fragments using g-TUBE (Covaris). The library was prepared based on the 20 kb PacBio sample preparation protocol. Size selection was performed using BluePippin (Sage Science), according to the manufacturer’s protocol, and that library was sequenced using the P6/C4 chemistry on four single-molecule real-time (SMRT) cells (two with BluePippin and two without) and a 240 min collection time. These continuous long read data were *de novo* assembled using the PacBio hierarchical genome assembly process HGAP3.0 with default parameters. Resulting assemblies were processed using Gepard [24] to identify overlapping regions at the ends, and then trimmed [25]; this was done for both chromosomes and plasmids. Potential SNPs/indels were corrected with Pilon v1.18 [26] using paired-end short-read data (2×250 bp) obtained from the Illumina MiSeq platform (averaging 232× coverage), then mapped to the reference sequences via Bowtie2 v2.2.9 [27].

### PT analysis pipeline

Because the data collected through GenomeTrakr are primarily used for phylogenetic clustering, our analysis of the data submitted by the laboratories was designed accordingly, resulting in an internal PT analysis pipeline that assessed relevant data-quality metrics. For each PT isolate, reference-based alignment and variant calling was performed on the data submitted by the PT-participating laboratories using the Center for Food Safety and Applied Nutrition (CFSAN) SNP Pipeline v0.8.1 [3] and complete reference genomes as described previously; the default parameters were used, except for a minimum MAPQ of 20 (SamtoolsMpileup_ExtraParams='-q 20 -Q 13'). Sequencing metrics were retrieved from the metrics.tsv results file. The SNPs reported by the FDA PT analysis pipeline are the Phase2 filtered SNPs designated as Phase2_Preserved_SNPs. Sequencing run quality was assessed using FastQC v0.11.4 [28]. Assembly quality was assessed using the SPAdes Genome Assembler v3.8.0 [29] and quast v3.0 [30]. Correlation analyses were performed by calculating Pearson’s *r* and Spearman’s rho rank correlation coefficients with pairwise deletion using the rcorr function of the Hmisc R library [31]. In order to correct for multiple comparisons with positive dependency, *P* values were adjusted using the Benjamini–Hochberg procedure [32].

### Public data submission

Raw sequence data collected as part of the 2015 PT exercise were deposited in the NCBI’s SRA (https://www.ncbi.nlm.nih.gov/sra) within the BioProject PRJNA386310 (Table S1). Complete annotated assemblies for each of the eight distributed PT strains were also deposited at GenBank; corresponding accession numbers and Bioproject ID numbers are indicated in Table 2.

## Results

Twenty-one of the 26 GTPT participants followed the protocol AND submitted raw fastq files for at least six of the eight PT strains, along with basic sequencing run metrics such as ‘cluster density’ and ‘reads passing filter’. Genomic data submitted by each of the 21 laboratories were analysed using the FDA-CFSAN internal PT analysis pipeline, comparing each of the submissions against our reference genomes, in order to evaluate each submitted sequence for possible sample mix-ups, sequence quality, depth of coverage, assembly quality and presence of SNPs. Laboratory-specific reports were generated to summarize the PT analysis results for each laboratory, specifying where their submission fell in relation to the other participants. For this paper, we provide the analytical results of the entire exercise in Table S1; from these results, we developed further correlation studies and graphics.

### Sequence analysis

The first evaluation was to check for sample mix-ups, which was done by mapping the submitted reads against the corresponding reference genome. Out of 203 submitted PT strains, we found only one annotation error (a sample swap) affecting two isolates. These were correctly re-labelled by us for all subsequent analyses.

**Fig. 1.**
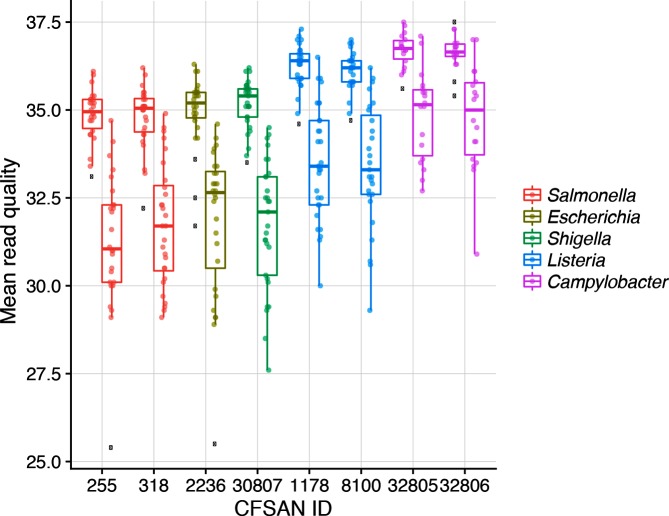
Box and whisker plots summarizing the mean read quality across Read1 and Read2 for each of the eight PT strains. The box defines the median value, as well as the lower and upper quartiles (25 and 75 %). The whiskers extend to the most extreme data point that is no more than 2.5 times the interquartile range from the median. Outliers are shown as black dots. The colours are genus specific.

We then visualized sequence quality for each of the eight PT isolates by plotting the mean Q score for each read, forward (R1) and reverse (R2). R2 was consistently lower quality than R1 for each of the isolates, by 3–4 Q scores (Fig. 1). Although two samples had R2 Q scores below Q28, GenomeTrakr’s minimum threshold for quality, the mean Q scores from the remaining sequences submitted far exceeded this threshold. However, while many of the metrics in this analysis were expected to reflect taxonomic differences, due to physical characteristics such as differences in genome sizes across the different bacteria, we observed an unexpected statistically significant effect of taxonomy on Q score (all adjusted *P* values <0.0015 with Tukey multiple comparison of means), with the *Campylobacter* isolates producing the highest quality reads and the four *Enterobacteriaceae* isolates, or enterics, producing the lowest quality.

**Fig. 2.**
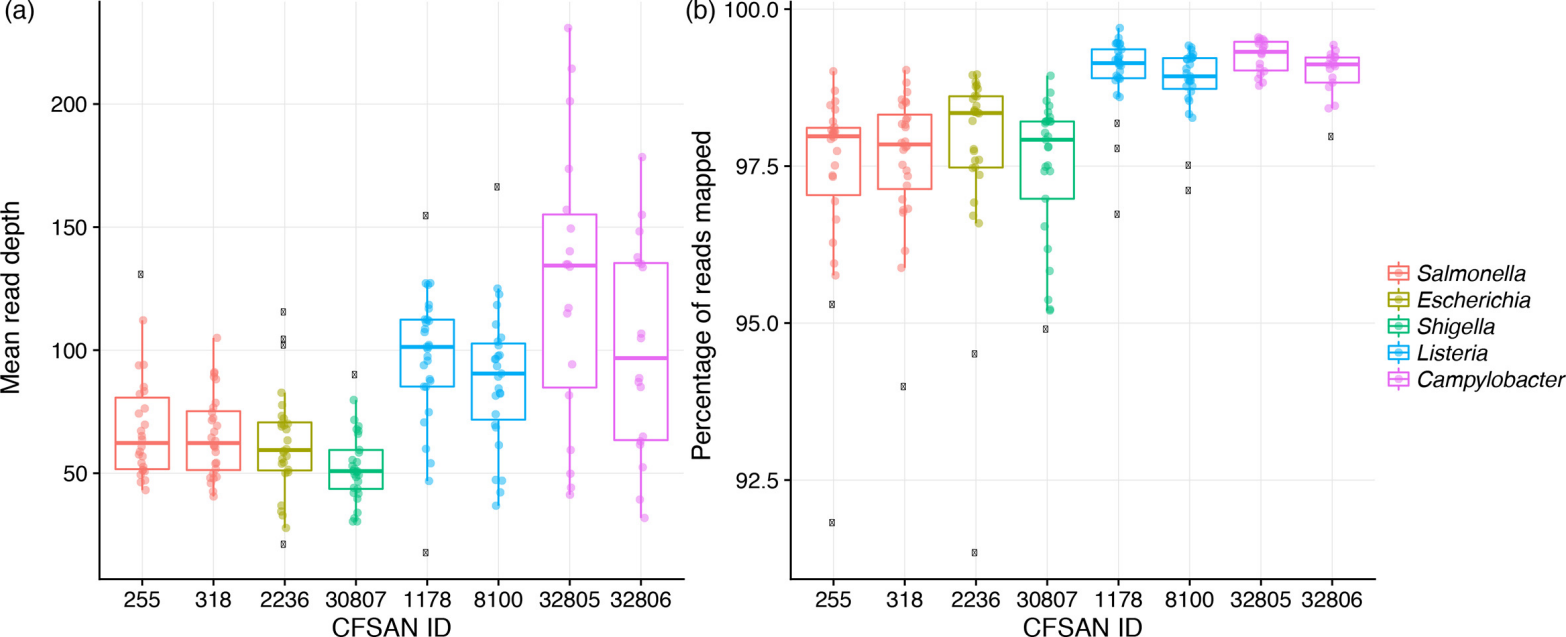
Box and whisker plots summarizing two different coverage statistics: (a) mean read depth and (b) percentage of reads mapped to the reference. The box defines the median value, as well as the lower and upper quartiles (25 and 75 %). The whiskers extend to the most extreme data point that is no more than 2.5 times the interquartile range from the median. Outliers are shown as black dots. The colours are genus specific.

Mean genome coverage or read depth (i.e. the depth of raw reads mapped back to the reference genome) varied according to genome size (Fig. 2a). The coverage for *Salmonella enterica*, *E. coli* and *Shigella sonnei* averaged in the 60× range, *L. monocytogenes* in the 90–100× range, and the campylobacters in the 100–150× range. However, there was negligible taxonomic difference for the percentage of reads that mapped back to the reference. All eight isolates ranged in the upper 97–99 % (Fig. 2b), with the enterics hovering at the lower end of this range.

We counted the number of SNPs in each submitted PT isolate compared to each respective reference genome. In 73 % of the PT submissions, zero SNPs were detected (149/203) (Table 3). When SNPS were identified these ranged from 1 to 4 per isolate, many of which, are shared by multiple isolates (Table S3). Our analyses indicated that there was one consistent SNP difference, or conserved mutation, between the isolate distributed for *C. jejuni* CFSAN032806 and its reference genome.

**Table 3.** Summary of SNPs detected in the raw PT data when compared to each respective reference genome Five columns are included (0–4 SNPs) with counts of genomes submitted for that number of SNPs.

**Organism**	**ID**	**0 SNPs**	**1 SNP**	**2 SNPs**	**3 SNPs**	**4 SNPs**
*Salmonella enterica* ser. Montevideo	CFSAN000255	20	6	0	0	0
*Salmonella enterica* ser. Heidelberg	CFSAN000318	22	4	2	0	0
*Listeria monocytogenes*	CFSAN001178	16	9	1	2	1
*Escherichia coli*	CFSAN002236	22	2	2	1	1
*Listeria monocytogenes*	CFSAN008100	24	1	2	0	0
*Shigella sonnei*	CFSAN030807	28	1	0	0	0
*Campylobacter coli*	CFSAN032805	17	1	0	0	0
*Campylobacter jejuni*	CFSAN032806	0	16	2	0	0

**Fig. 3.**
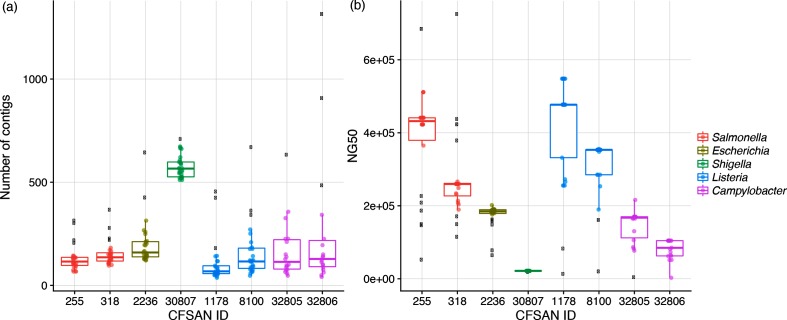
Box and whisker plots summarizing two different assembly statistics: (a) number of contigs and (b) NG50, a measure of contig length. The box defines the median value, as well as the lower and upper quartiles (25 and 75 %). The whiskers extend to the most extreme data point that is no more than 2.5 times the interquartile range from the median. Outliers are shown as black dots. The colours are genus specific.

Assembly quality is the last metric summarized in our PT analysis. The number of contigs varied across the eight PT isolates (Fig. 3a). For example, *Shigella sonnei* assemblies averaged over 500 contigs, whereas the assemblies of other seven isolates averaged in the 100 contig range. Along with the number of contigs, we also calculated the NG50 for each assembly, a measure of the length of the median-sized contig (Fig. 3b). The summary values varied for this statistic, some with narrow (*Shigella sonnei*) variation versus some with wide variation (*L. monocytogenes*). The two *Salmonella enterica* strains and two *L. monocytogenes* strains had the highest NG50 scores, while *Shigella sonnei* had the lowest. The Nextera XT library preparation kit resulted in a library with insert sizes averaging in the 300–400 bp range across all eight PT strains (Fig. S1).

### Run metrics

**Fig. 4.**
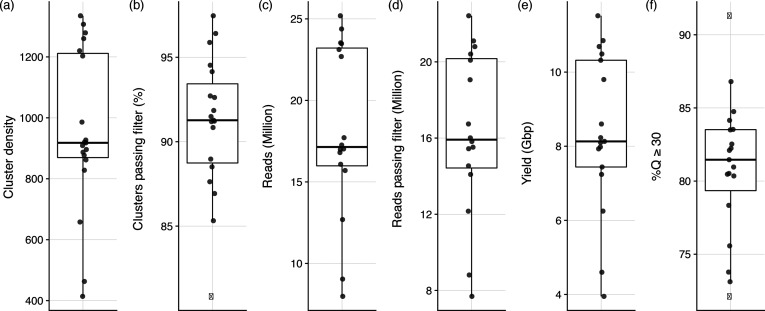
Box and whisker plots summarizing six different MiSeq run metrics reported by the participating laboratories: (a) cluster density, (b) clusters passing filter, (c) number of reads collected, (d) per cent of reads passing filter, (e) total data yield in Gbp, and (f) percentage of Q scores greater or equal to Q30. The box defines the median value, as well as the lower and upper quartiles (25 and 75 %). The whiskers extend to the most extreme data point that is no more than 2.5 times the interquartile range from the median. Outliers are shown as black dots. Abbreviations for isolate metrics: Reads_M, number of sequencing reads passing filter for a given isolate; SeqLength, range of sequencing read lengths; MeanR1, Q score representing the mean of the mean read quality of R1 (forward) reads; MeanR2, Q score representing the mean of the mean read quality of R2 (reverse) reads; PercMapped, percentage of reads that could be mapped to the reference genome; MeanDepth, mean depth (coverage) of reads mapped to the reference genome; SNPs, SNPs reported by the CFSAN SNP Pipeline; MeanInsert, mean insert size, defined as the length of the sequence between the adapters; GenomeFraction, total number of aligned bases in the reference, divided by the genome size (a base in the reference genome is counted as aligned if at least one contig has an alignment to the base; contigs from repeat regions may map to multiple places and, thus, may be counted multiple times); NG50, contig length such that using equal or longer length contigs produces x% of the length of the reference genome, allowing for comparisons between different genomes (larger NG50 values generally correlate with a higher quality assembly); Contigs, total number of contigs in the assembly (fewer contigs generally correlate with a higher quality assembly). *Abbreviations for run metrics*: ClusterDensity, density of clusters (K mm^−2^); PercClustersPF, percentage of clusters which passed filtering; Reads_M, number of reads (clusters) in millions; ReadsPF_M, number of reads (clusters) that passed filtering (millions); Yield, number of gigabases that passed filtering; Q30, percentage of bases with a Q score ≥30.

Although not officially part of the 2015 PT analysis report, we summarized metrics surrounding the corresponding MiSeq run, which were provided by most of the laboratories (Table S3). On average, cluster density was 955 K mm^−2^ (Fig. 4a), 91 % of clusters passed filter (Fig. 4b), 18 million reads were collected of which 16 million (91 %) passed filter (Fig. 4c, d), 91 % of reads passed filter, 8.4 Gbp of data were collected for each run (Fig. 4e), and 81 % of bases had a quality (Q) score of Q30 or above (Fig. 4f).

### Correlation statistics

**Fig. 5.**
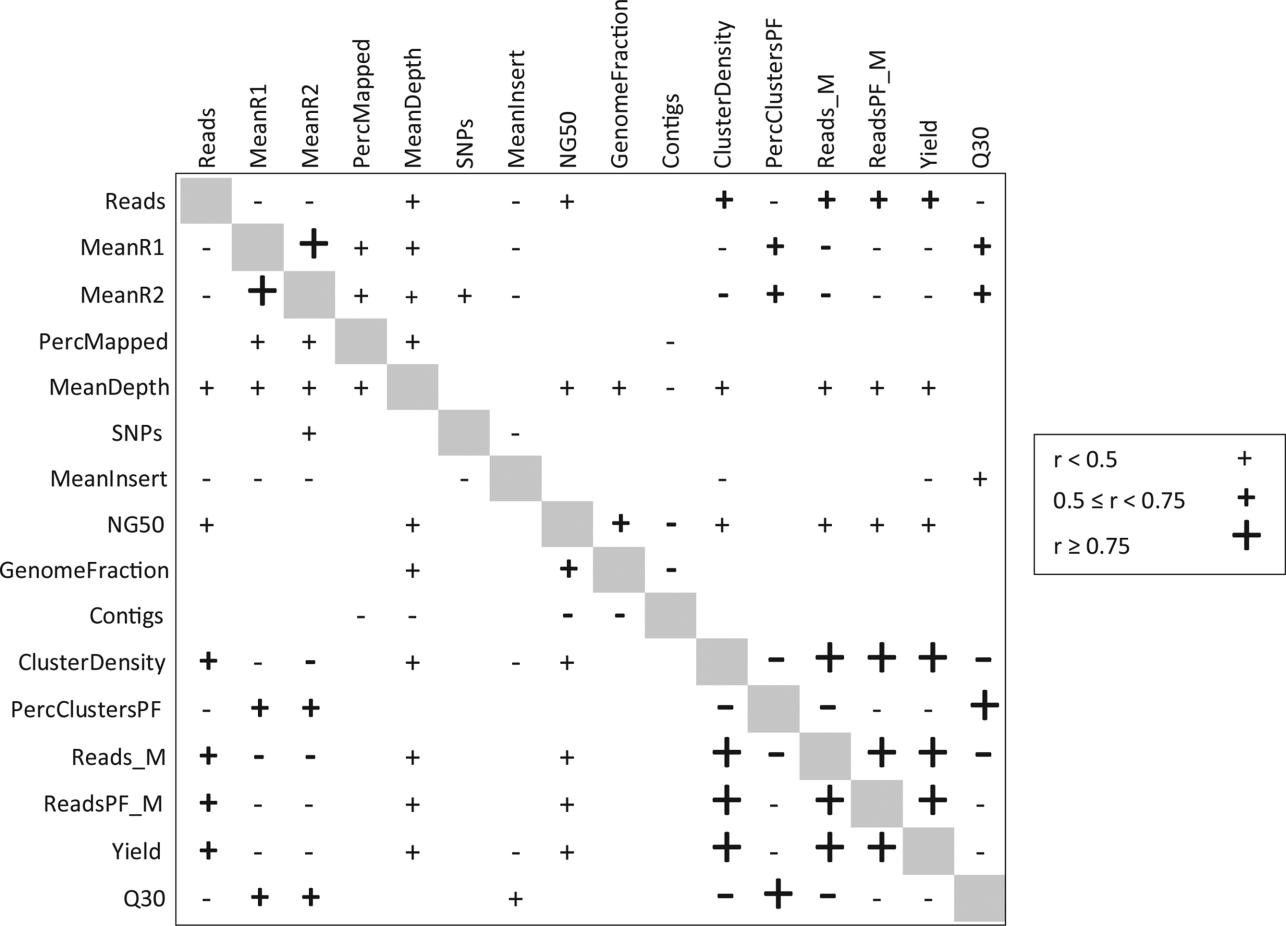
Pearson correlation table of run metrics and summary statistics. Significant positive and negative correlations are represented as + and −, respectively. The size of the symbols and font boldness represent the degree of correlation.

One of the expected benefits of compiling and analysing the GTPT dataset was the ability to examine relationships between run- and sequence-level metrics, specifically to test assumptions regarding the possible benefits of optimizing laboratory-based metrics, such as insert size and cluster density. Given that linear correlations were deemed most useful in predicting the outcome of purposeful modifications to one of the variables (monotonic to a lesser degree), we calculated both Pearson’s *r* (a measure of linear correlation) and Spearman’s rho (assumes a monotonic correlation) for each run- and sequence-level metric One of the expected benefits of compiling and analysing the GTPT dataset was the ability to examine relationships between run- and sequence-level metrics, specifically to test assumptions regarding the possible benefits of optimizing laboratory-based metrics such as insert size and cluster density and reported all significant correlations (*P≥*0.05) (Fig. 5) and Table S4, respectively). As a default, we are presenting the Pearson’s test (a more powerful statistical test and most appropriate for interval-scale data) but recognize many of these correlations might be non-linear and so included the Spearman’s test in the supplemental files for consideration. The Pearson’s test revealed that all of the run metrics were correlated with each other (Fig. 5), as seen in the lower right quadrant. Specifically, a strong positive correlation (*r* ≥0.75) was found between Cluster Density and number of Reads Collected, number of Reads Passing Filter and Overall Yield (Gbp collected). Conversely, a strong negative correlation was found between cluster density and clusters passing filter, percentage of reads passing filter and Q score. We saw medium strength correlations (0.5≥*r* <0.75) between run metrics and three read statistics: Reads per Isolate, Mean R1 Q Score and Mean R2 Q Score. R1 and R2 Q scores were positively correlated with percent of Clusters Passing Filter, percent of Reads Passing Filter and overall Q30 for the run, but negatively correlated with Cluster Density, number of Reads Collected and Overall Yield.

Fig. 5 is useful for examining one parameter in relation to all the others tested. For example, Mean Insert Length showed weak (*r*<0.5), but significant negative correlation with R1 and R2 Q Scores, Number of Reads, SNPs, Cluster Density, Overall Yield, and a weak positive correlation with the run quality metric (%≥Q30). Of our two metrics for assembly quality, NG50 (larger is generally better) showed a positive correlation with metrics involving the amount of data collected (number of Reads per Isolate, Mean Depth, Cluster Density, number of Reads per Run and Overall Run Yield), while the number of contigs (smaller is better) did not correlate with the amount of data collected, with the exception of Mean Depth. We noticed that although Cluster Density was positively correlated with metrics related to the overall yield and negatively correlated with metrics related to base quality, there were no negative correlations with downstream analysis metrics including percentage of Reads Mapped and number of contigs. Instead, there was a weak positive correlation with the NG50 assembly quality metric. Also of interest, the percentage of reads mapped did not show correlation with any of the run metrics, including the run quality metric (%≥Q30).

## Discussion

As growing numbers of public-health laboratories use WGS technology to support enforcement decisions, it is essential to have confidence in the quality of the data being used and the downstream data analyses that guide these decisions. Routine proficiency tests, such as the one described here and the one used by the GMI group have an important role in ensuring the validity of both data and procedures. Overall, this exercise revealed the degree of variation which should be expected in sequence data produced across a diverse network of laboratories. Our interpretation is that Illumina MiSeq sequence data collected for the same set of strains across 21 different laboratories exhibited high reproducibility, while revealing a narrow range of technical and biological variance within the clones sent out for sequencing. The numbers of SNPs reported for sequencing runs of the same isolates across multiple laboratories support the robustness of our cluster analysis pipeline [3] in that each individual isolate cultured and resequenced multiple times in multiple places are all easily identifiable as originating from the same source. The quality and coverage metrics received from the participating laboratories generally far exceeded the existing GenomeTrakr QC thresholds that govern whether WGS submissions are acceptable for entry into the GenomeTrakr database (20× coverage and a mean quality score of Q28 or above on both R1 and R2).

From the perspective of the participating laboratories, this exercise provided valuable feedback, indicating the quality of their sequencing data in relation to the other laboratories that participated in the PT. For example, if one laboratory learned from this PT that they were achieving lower than average coverage, team members could go back through their laboratory preparation steps to determine a possible cause. It is also possible for results from this exercise to reveal that a particular sequencing machine is underperforming (quality or yield) and which may require servicing or optimization. Also, any facility that handles samples runs the risk of sample handling errors, estimated by one study to be an issue in almost 50 % of publications on human transcriptomics [33]. With this in mind, we designed our PT to detect mislabelling and/or sample switches. Out of the 203 total submissions, we identified one sample swap, which caused two PT samples to be identified incorrectly – a handling-caused error rate of ~1 %. By identifying sample handling errors under these controlled conditions, laboratories are able to strengthen their local SOPs by adding checks that reduce or eliminate the likelihood of labelling errors during sample processing.

### Interesting findings

This project provided some interesting correlations that ran counter to our expectations of how upstream decisions affect downstream analytic outcomes. For example, cluster density is expected to correlate positively with yield-based metrics and negatively with quality-based metrics [34]. In other words, as more DNA is loaded onto the chip, more data are collected, but overclustering causes a *decrease* in quality of those data. Although we observed this to be true, we also found that our downstream analysis metrics, like assembly, were not inversely correlated with cluster density and that the NG50 metric shows positive correlation. While we do not believe there to be zero correlation, the implication of this finding may be of great interest to laboratories and networks attempting to reduce the cost of sequencing by multiplexing larger quantities of isolates. Many of the laboratories in this exercise reported overclustering (Table S2), while maintaining acceptable mapping and assembly metrics. Over the 414–1335 range of cluster densities observed in this exercise, we saw no decrease in usable data when Q30 is above 75 %. So, although overclustering reduces the overall quality of the run, as long as the minimum Q score is met for the run the increased yield may be of greater benefit to the user. We have also held the assumption that maximizing insert length for paired-end sequencing should help improve the assembly. However, we did not see an effect within the 250 to 550 bp range of insert sizes in our PT exercise. Even though it makes sense to want larger insert sizes, this parameter does not appear to impact downstream analyses within our dataset (Fig. 5). There is a slight inverse correlation between insert size and number of reads, mean R1 and R2 Q scores, number of SNPs, cluster density and overall yield for the run. In other words, as more library is added to the run, the mean insert size gets smaller. Perhaps smaller insert sizes are preferentially sequenced in overloaded runs? However, if there is no impact on downstream coverage or assembly, then there is minimal advantage in optimizing insert sizes for the Nextera XT prep given our >50 ×/isolate. Percentage of mapped reads is a metric thought to be influenced the quality of the run and, thus, run metrics, but within the 95–99 % range reported in this exercise we didn’t see any linear correlation here. In this exercise, we didn't see any relationship by Pearson's test for linear correlation. Spearman correlation, a measure of the monotonic relationship between variables, *did* report positive and negative correlations here, so perhaps the quality of the run does influence the percentage of mapped reads, but the relationship is non-linear (Table S4).

Another interesting pattern emerged from the sequence quality assessment – a significant difference in read quality depending on taxonomy. Fig. 1 shows three different categories of quality. The enterics, *Salmonella enterica, E. coli* and *Shigella sonnei* (*Gammaprotobacteria*), all have a similar pattern, the two *L. monocytogenes* strains (*Firmicutes*) have slightly higher quality and finally the two *Campylobacter* strains (*Epsilonprotobacteria*) have the highest quality. There are several differences between these three groups. (1) The bacterial taxonomy: each respective taxonomic order holds its own unique phylogenetic position. (2) They each have different culturing steps: the enterics are grown in trypticase soy broth (TSB), *L. monocytogenes* is grown in TSB and the *Campylobacter* strains are grown on blood agar plates, with cultures taken from the plate and resuspended in water. (3) While the DNA extraction is uniform across these taxa, there is an extra enzymatic lysis step for *L. monocytogenes* prior to the DNA isolation. (4) Genome size and G+C content also differ across these three categories: enterics (*Enterobacteriaceae*) ~4.7 MB, 50 mol% G+C; *L. monocytogenes* 2.9 MB, 38mol% G+C; and *Campylobacter* strains 1.8 MB, 30 mol% G+C. Even through these last differences appear to track the improvement in sequence quality (smaller genomes and lower G+C content have higher quality sequence), we cannot think of a causal reason for this. There are reports of G+C bias affecting genome coverage due to amplification biases [35–37], but none that have found a G+C bias affecting actual sequence quality or the PHRED scores themselves. Our own correlation data reveals a positive correlation between read quality and a few upstream variables affecting the entire run: clusters passing filter, reads passing filter and the run quality metric (%≥Q30) (Fig. 5), none of which would be taxon specific. It is possible that sample preparation methods affect sequence quality [37], for example the culturing/lysis methods differ for these three species, which might influence DNA quality and in turn influence sequence quality. Sequence quality has an impact on downstream processes, such as read mapping, depth of coverage, SNPs detected and mean insert size (Fig. 5), so understanding the source of this variation would be very valuable.

### Dataset importance

In this work, we make available two important datasets: (1) a table of metrics summarized in the PT test (Table S1), and (2) the raw data gathered in this exercise. The table serves as a valuable resource for laboratories that want to replicate this PT test in their own environment and then analyse their results in context of data collected during this exercise. The figures and tables presented in this paper were all derived from this summary table. If laboratories are interested in setting pass/fail thresholds, we suggest identifying the most important metrics for the laboratory, then setting thresholds at the appropriate edge of the box plots (the lower and upper quartiles) or the appropriate edge of the whiskers (the most extreme data point that is no more than 2.5 times the interquartile range from the median). The second type of data, the actual raw sequence data collected in this exercise, is a rare release of raw data from a WGS PT exercise. As more laboratories adopt the rigorous WGS CLIA standard [19], it will be ever more important to share the raw data from large proficiency exercises such as this one to provide an additional layer of validation. Combing through these datasets enables us, as regulatory bodies, to understand laboratory-to-laboratory variation, to build informed quality assurance/QC thresholds around the collection of these important data, and to ensure proper validation for each NGS technology and accompanying analyses. It is obvious to see how these quality assurance steps are important for public health and proper disease surveillance, but they also provide important transparency for the industries that are most effected by regulatory action (recalls, seizures, injunctions, etc.). For past technologies (i.e. PFGE), industry would have to issue a Freedom of Information Act (FOIA) request to access data collected from this type of exercise. Making our WGS PT data public follows the ‘open data’ model of GenomeTrakr and overall movement that USA public-health agencies are striving to meet. From this exercise, we confirmed that WGS is reliable and consistent for use in microbial surveillance in that identical samples can be identified as such. This point is critical for our downstream clustering pipeline [3] that relies on data collection consistency for drawing conclusions about which isolates are where at what time. We also hope that standardized datasets like this will help provide baselines for academic research questions.

## Supplementary Data

Supplementary File 1
