# Supplementary material for: GenomeTrakr proficiency testing for foodborne pathogen surveillance: an exercise from 2015
Source: Microb Genom. 2018 Jun 15;4(7):e000185. doi: 10.1099/mgen.0.000185 (PMC6113870; doi:10.1099/mgen.0.000185)
Supplement: Supplementary File 1 [file mgen-4-185-s001.pdf]

Supplemental Figure 1. Insert sizes

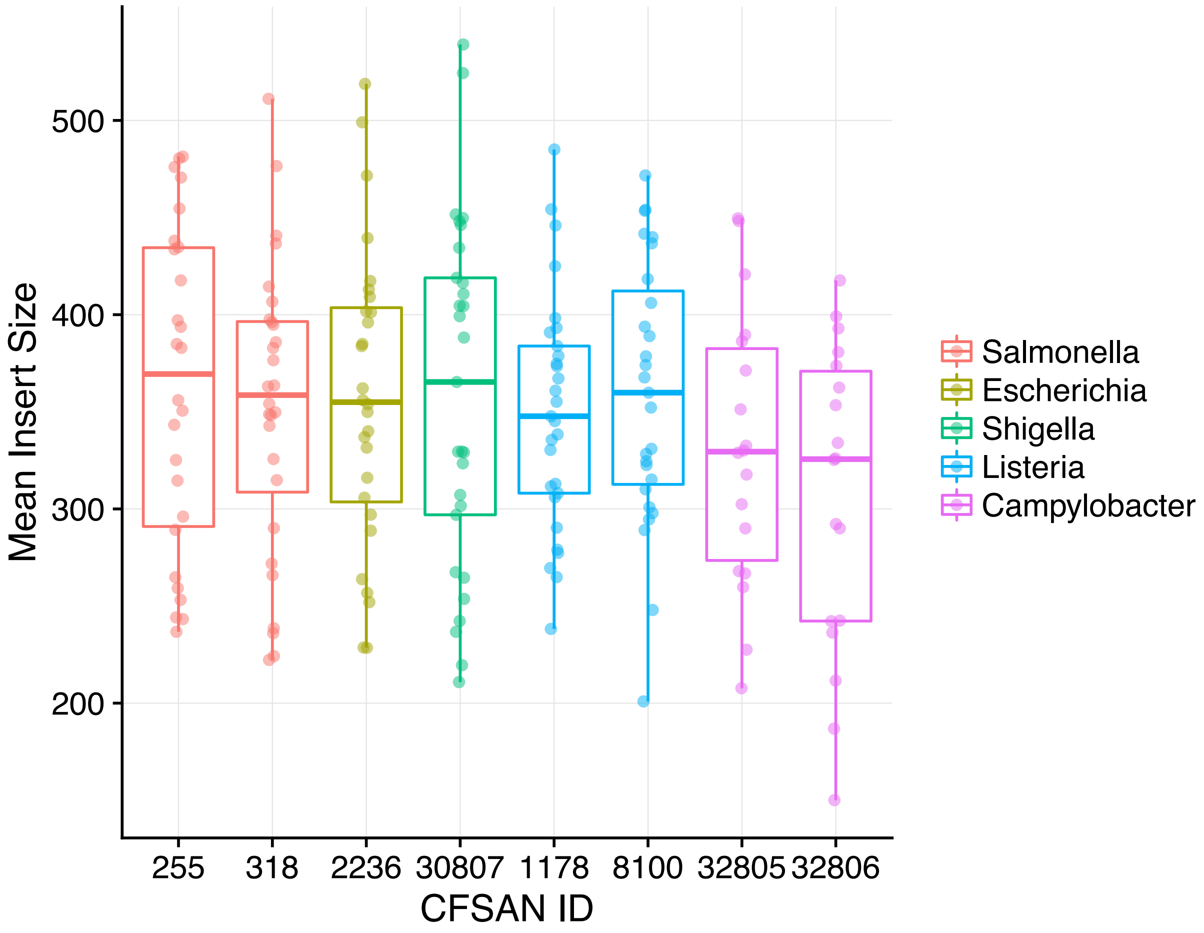

Supplemental Table 1. Raw data and summary statistics for each individual sequence in the exercise. NCBI SRA accessions included for access to the raw data.

					Run Metrics							Isolate Metrics									
Masked ID	Strain Name	Organism	NCBI BioSample	SRA Run	ClusterDensity	PercClustersPF	Reads_M	ReadsPF_M	Yield	Q30	SeqLength	Reads	MeanR1	MeanR2	PercMapped	MeanDepth	SNPs	MeanInsert	NG50	GenomeFraction	Contigs
1	CFSAN000255	Salmonella enterica subsp. enterica serovar Montevideo	SAMN07210937	SRR5805961	463	97.46	9.04	8.81	4.4	89.92	35-251	1781716	36	34.1	99.01	58.75	1	355.96	52111	98.678	218
1	CFSAN000318	Salmonella enterica subsp. enterica serovar Heidelberg	SAMN07210938	SRR5805705	463	97.46	9.04	8.81	4.4	89.92	35-251	2616190	36.2	34.4	98.83	74.74	0	325.67	170933	99.143	135
1	CFSAN001178	Listeria monocytogenes	SAMN07210944	SRR5805561	463	97.46	9.04	8.81	4.4	89.92	35-251	310516	37.3	35.9	99.33	17.64	1	378.76	13613	96.106	425
1	CFSAN002236	Escherichia coli	SAMN07210945	SRR5805563	463	97.46	9.04	8.81	4.4	89.92	35-251	1964546	36.1	33.9	98.96	58.45	0	353.95	64687	98.405	215
1	CFSAN008100	Listeria monocytogenes	SAMN07210931	SRR5805555	463	97.46	9.04	8.81	4.4	89.92	35-251	1008642	36.3	34.7	99.12	47.31	0	322.58	20306	97.123	342
1	CFSAN030807	Shigella sonnei	SAMN07210932	SRR5805956	463	97.46	9.04	8.81	4.4	89.92	35-251	1490828	36.1	34	98.94	48.46	0	416.42	18750	87.998	551
1	CFSAN032805	Campylobacter coli	SAMN07210933	SRR5805476	463	97.46	9.04	8.81	4.4	89.92	35-251	649628	37.5	37.1	99.55	44.15	0	267.97	4536	91.464	633
1	CFSAN032806	Campylobacter jejuni	SAMN07210930	SRR5805477	463	97.46	9.04	8.81	4.4	89.92	35-251	696852	37.5	37	99.24	39.31	1	242.51	2895	85.47	908
2	CFSAN000255	Salmonella enterica subsp. enterica serovar Montevideo	SAMN07210937	SRR5805962	NA	NA	NA	NA	NA	NA	35-251	1925082	35.2	30.9	98.06	67.19	0	384.86	422657	99.173	68
2	CFSAN000318	Salmonella enterica subsp. enterica serovar Heidelberg	SAMN07210938	SRR5805848	NA	NA	NA	NA	NA	NA	35-251	2298710	35	34.2	99.03	78.62	0	385.84	259788	99.216	99
2	CFSAN001178	Listeria monocytogenes	SAMN07210944	SRR5805564	NA	NA	NA	NA	NA	NA	35-251	2026694	36.3	34.7	99.54	111.2	1	367.18	548130	99.042	37
2	CFSAN002236	Escherichia coli	SAMN07210945	SRR5805562	NA	NA	NA	NA	NA	NA	35-251	1599342	35	32.7	98.84	54.36	0	396.01	186967	98.774	154
2	CFSAN008100	Listeria monocytogenes	SAMN07210931	SRR5805558	NA	NA	NA	NA	NA	NA	35-251	1609768	36.2	34.2	99.35	84.45	0	359.85	353282	98.634	47
2	CFSAN030807	Shigella sonnei	SAMN07210932	SRR5805971	NA	NA	NA	NA	NA	NA	35-251	1619846	35.2	31.5	98.21	50.91	0	399.21	21765	88.882	521
3	CFSAN000255	Salmonella enterica subsp. enterica serovar Montevideo	SAMN07210937	SRR5806408	NA	NA	NA	NA	NA	NA	35-251	1885178	35.4	32.7	97.35	47.13	0	289.24	440987	99.177	107
3	CFSAN000318	Salmonella enterica subsp. enterica serovar Heidelberg	SAMN07210938	SRR5805856	NA	NA	NA	NA	NA	NA	35-251	2112378	35.5	33	97.43	47.87	0	271.84	259854	99.196	96
3	CFSAN001178	Listeria monocytogenes	SAMN07210944	SRR5805579	NA	NA	NA	NA	NA	NA	35-251	1767856	36.3	32.5	98.89	87.5	0	345.21	476905	99.046	52
3	CFSAN002236	Escherichia coli	SAMN07210945	SRR5805581	NA	NA	NA	NA	NA	NA	35-251	1111480	35.3	31.2	97.47	32.86	0	383.83	186967	98.783	127
3	CFSAN008100	Listeria monocytogenes	SAMN07210931	SRR5805588	NA	NA	NA	NA	NA	NA	35-251	2360694	36.5	32.4	98.27	96.33	0	294.59	353282	98.44	66
3	CFSAN030807	Shigella sonnei	SAMN07210932	SRR5806406	NA	NA	NA	NA	NA	NA	35-251	1685838	35.7	31.2	96.54	39.66	0	329.08	21765	89.07	552
3	CFSAN032805	Campylobacter coli	SAMN07210933	SRR5805517	NA	NA	NA	NA	NA	NA	35-251	2102226	36.6	34	99.34	173.67	0	330.06	169981	98.913	88
3	CFSAN032806	Campylobacter jejuni	SAMN07210930	SRR5805512	NA	NA	NA	NA	NA	NA	35-251	1631928	36.6	33.6	99.15	137.79	1	373.68	104440	97.221	88
4	CFSAN000255	Salmonella enterica subsp. enterica serovar Montevideo	SAMN07210937	SRR5806411	887	90.84	16.99	15.44	7.93	84.15	35-251	1372822	34.8	30	97.33	51.36	0	454.63	511299	99.183	110
4	CFSAN000318	Salmonella enterica subsp. enterica serovar Heidelberg	SAMN07210938	SRR5805855	887	90.84	16.99	15.44	7.93	84.15	35-251	1790644	35	31.7	98.24	61.04	0	414.43	233832	99.215	144
4	CFSAN001178	Listeria monocytogenes	SAMN07210944	SRR5805857	887	90.84	16.99	15.44	7.93	84.15	35-251	2000512	36.4	33.2	99.1	112.41	1	393.21	477130	98.901	118
4	CFSAN002236	Escherichia coli	SAMN07210945	SRR5805584	887	90.84	16.99	15.44	7.93	84.15	35-251	2262594	35.4	29.3	96.59	73.27	0	412.96	190130	98.748	211
4	CFSAN008100	Listeria monocytogenes	SAMN07210931	SRR5805853	887	90.84	16.99	15.44	7.93	84.15	35-251	1802820	36.4	33.7	99.21	103.43	0	418.34	353282	98.541	87
4	CFSAN030807	Shigella sonnei	SAMN07210932	SRR5806409	887	90.84	16.99	15.44	7.93	84.15	35-251	2116716	35.4	30.1	97.42	67.85	0	434.43	21765	88.893	621
4	CFSAN032805	Campylobacter coli	SAMN07210933	SRR5805521	887	90.84	16.99	15.44	7.93	84.15	35-251	2555182	36.8	35.5	99.49	214.38	0	332.53	169981	98.895	93
4	CFSAN032806	Campylobacter jejuni	SAMN07210930	SRR5805514	887	90.84	16.99	15.44	7.93	84.15	35-251	1689236	36.7	35.5	99.43	155	1	399.09	104440	97.216	98
5	CFSAN000255	Salmonella enterica subsp. enterica serovar Montevideo	SAMN07210937	SRR5806430	909	91.22	17.02	15.52	7.98	83.53	35-251	1735198	35.3	31	97.93	65.12	0	438.06	511299	99.172	134
5	CFSAN000318	Salmonella enterica subsp. enterica serovar Heidelberg	SAMN07210938	SRR5806438	909	91.22	17.02	15.52	7.98	83.53	35-251	1828176	35.3	30.9	97.8	64.47	1	440.66	378695	99.218	171
5	CFSAN001178	Listeria monocytogenes	SAMN07210944	SRR5805873	909	91.22	17.02	15.52	7.98	83.53	35-251	1870882	36.4	31.3	98.18	108.6	0	424.95	548130	99.046	117
5	CFSAN002236	Escherichia coli	SAMN07210945	SRR5805591	909	91.22	17.02	15.52	7.98	83.53	35-251	1671228	35.5	32.9	98.77	59.02	0	439.42	189159	98.762	202
5	CFSAN008100	Listeria monocytogenes	SAMN07210931	SRR5805870	909	91.22	17.02	15.52	7.98	83.53	35-251	1606654	36.4	34.4	99.21	93.52	0	436.73	353282	98.541	184
5	CFSAN030807	Shigella sonnei	SAMN07210932	SRR5806444	909	91.22	17.02	15.52	7.98	83.53	35-251	1662266	35.5	32.1	98.21	55.37	0	449.75	21765	88.878	568
5	CFSAN032805	Campylobacter coli	SAMN07210933	SRR5805540	909	91.22	17.02	15.52	7.98	83.53	35-251	1648470	36.7	35.1	99.39	157.05	0	386.3	169981	98.915	225
5	CFSAN032806	Campylobacter jejuni	SAMN07210930	SRR5805539	909	91.22	17.02	15.52	7.98	83.53	35-251	1168714	36.6	33.5	98.91	104.89	1	417.63	104440	97.23	141
6	CFSAN000255	Salmonella enterica subsp. enterica serovar Montevideo	SAMN07210937	SRR5911613	1203	88.51	22.69	20.09	10.32	75.57	35-251	3356280	34.7	29.3	96.65	93.84	0	325.15	422825	99.192	302
6	CFSAN000255	Salmonella enterica subsp. enterica serovar Montevideo	SAMN07210937	SRR5911152	1203	88.51	22.69	20.09	10.32	75.57	35-251	2469650	35.1	30.5	96.28	54.17	0	253.16	441276	99.178	314
6	CFSAN000318	Salmonella enterica subsp. enterica serovar Heidelberg	SAMN07210938	SRR5910649	1335	88.97	25.19	22.42	11.52	73.12	35-251	3007268	35.4	31.7	96.8	60.58	0	236	259788	99.218	367
6	CFSAN000318	Salmonella enterica subsp. enterica serovar Heidelberg	SAMN07210938	SRR5910647	1203	88.51	22.69	20.09	10.32	75.57	35-251	3095464	35.1	29.1	93.99	54.24	0	224.24	259788	99.207	278
6	CFSAN001178	Listeria monocytogenes	SAMN07210944	SRR5910641	1203	88.51	22.69	20.09	10.32	75.57	35-251	2852070	35.9	31.6	97.78	126.56	1	311.6	477130	99.056	455
6	CFSAN001178	Listeria monocytogenes	SAMN07210944	SRR5910644	1203	88.51	22.69	20.09	10.32	75.57	35-251	2927350	36	30	96.73	111.9	0	277.34	331773	99.039	180
6	CFSAN002236	Escherichia coli	SAMN07210945	SRR5910643	1203	88.51	22.69	20.09	10.32	75.57	35-251	2893216	34.8	25.5	91.34	67.84	0	336.95	190130	98.767	267
6	CFSAN002236	Escherichia coli	SAMN07210945	SRR5912122	1203	88.51	22.69	20.09	10.32	75.57	35-251	4052296	35.4	32.5	97.48	77.69	0	228.64	186967	98.774	426
6	CFSAN008100	Listeria monocytogenes	SAMN07210931	SRR5911615	1203	88.51	22.69	20.09	10.32	75.57	35-251	2948494	36	30.6	97.51	125.05	0	310.12	353282	98.61	362
6	CFSAN008100	Listeria monocytogenes	SAMN07210931	SRR5911618	1203	88.51	22.69	20.09	10.32	75.57	35-251	2029442	35.7	29.3	97.11	105.1	0	388.98	353282	98.575	271
6	CFSAN030807	Shigella sonnei	SAMN07210932	SRR5910645	1335	88.97	25.19	22.42	11.52	73.12	35-251	2712880	35.6	32.5	97.42	50.48	0	236.73	21425	89.325	710
6	CFSAN030807	Shigella sonnei	SAMN07210932	SRR5911619	1335	88.97	25.19	22.42	11.52	73.12	35-251	3073864	35.5	31.3	96.18	49.5	0	210.84	21425	89.523	669
6	CFSAN030807	Shigella sonnei	SAMN07210932	SRR5912124	1203	88.51	22.69	20.09	10.32	75.57	35-251	1925424	35.1	29.4	95.83	42.04	0	301.56	21940	89.207	662
6	CFSAN032805	Campylobacter coli	SAMN07210933	SRR5912123	1203	88.51	22.69	20.09	10.32	75.57	35-251	2191054	36.2	33.5	98.78	201.13	0	371.24	169981	98.903	357
6	CFSAN032805	Campylobacter coli	SAMN07210933	SRR5912126	1203	88.51	22.69	20.09	10.32	75.57	35-251	2934246	36.1	33.6	98.96	230.91	0	317.68	169981	98.881	326
6	CFSAN032806	Campylobacter jejuni	SAMN07210930	SRR5912125	1203	88.51	22.69	20.09	10.32	75.57	35-251	2761352	36.5	33.3	97.97	133.69	1	211.57	81691	97.012	342
6	CFSAN032806	Campylobacter jejuni	SAMN07210930	SRR5912127	1203	88.51	22.69	20.09	10.32	75.57	35-251	2407226	36.3	33.4	99.09	178.45	1	325.26	104440	97.073	41
7	CFSAN000255	Salmonella enterica subsp. enterica serovar Montevideo	SAMN07210937	SRR2544685	927	94.53	17.71	16.74	8.6	86.8	35-251	1439588	35.3	31.6	98.12	57.59	0	470.6	684920	99.196	136
7	CFSAN000318	Salmonella enterica subsp. enterica serovar Heidelberg	SAMN07210938	SRR2544686	927	94.53	17.71	16.74	8.6	86.8	35-251	1957038	35.7	33.9	98.68	58.63	1	348.18	423434	99.153	220
7	CFSAN001178	Listeria monocytogenes	SAMN07210944	SRR5805548	927	94.53	17.71	16.74	8.6	86.8	35-251	2797020	37	35.2	99.46	127.25	0	305.99	476905	99.046	61
7	CFSAN001178	Listeria monocytogenes	SAMN07210944	SRR2544687	927	94.53	17.71	16.74	8.6	86.8	35-251	1673618	36.9	35.8	99.7	95.73	0	390.87	477130	99.048	57
7	CFSAN002236	Escherichia coli	SAMN07210945	SRR5805546	927	94.53	17.71	16.74	8.6	86.8	35-251	1756368	35.7	33.4	98.62	59.81	1	409.15	201815	98.723	644
7	CFSAN008100	Listeria monocytogenes	SAMN07210931	SRR5805552	927	94.53	17.71	16.74	8.6	86.8	35-251	2255378	37	36.2	99.42	97.98	0	300.84	353282	98.625	177
7	CFSAN030807	Shigella sonnei	SAMN07210932	SRR5805954	927	94.53	17.71	16.74	8.6	86.8	35-251	1502124	35.7	33.1	98.67	50.86	0	446.18	21943	92.082	664
8	CFSAN000255	Salmonella enterica subsp. enterica serovar Montevideo	SAMN07210937	SRR5806399	NA	NA	NA	NA	NA	NA	35-251	1966780	33.1	25.4	91.82	56.85	0	393.73	208505	99.071	132
8	CFSAN000318	Salmonella enterica subsp. enterica serovar Heidelberg	SAMN07210938	SRR5805858	NA	NA	NA	NA	NA	NA	35-251	2253778	32.2	30.2	97.52	71.53	2	394.83	207744	99.216	105
8	CFSAN001178	Listeria monocytogenes	SAMN07210944	SRR5805863	NA	NA	NA	NA	NA	NA	35-251	1239776	34.6	31.6	98.9	59.91	4	330.37	82586	98.934	95
8	CFSAN002236	Escherichia coli	SAMN07210945	SRR5805583	NA	NA	NA	NA	NA	NA	35-251	2927994	32.5	32.4	97.59	72.25	3	305.78	179966	98.237	194
8	CFSAN008100	Listeria monocytogenes	SAMN07210931	SRR5805859	NA	NA	NA	NA	NA	NA	35-251	1475914	34.9	31.8	98.86	73.95	0	352.2	253419	98.603	92
8	CFSAN030807	Shigella sonnei	SAMN07210932	SRR5806419	NA	NA	NA	NA	NA	NA	35-251	2360568	33.5	27.6	95.2	67.22	0	418.96	20031	88.302	587

9	CFSAN000255	Salmonella enterica subsp. enterica serovar Montevideo	SAMN07210937	SRR5806402	862	87.63	16.08	14.09	7.24	78.34	35-251	1462788	33.4	31	97.51	46.38	1	397.09	146900	99.081	111
9	CFSAN000318	Salmonella enterica subsp. enterica serovar Heidelberg	SAMN07210938	SRR5805864	862	87.63	16.08	14.09	7.24	78.34	35-251	1626256	33.2	31.1	97.82	49.94	1	382.86	149654	99.123	182
9	CFSAN001178	Listeria monocytogenes	SAMN07210944	SRR5805582	862	87.63	16.08	14.09	7.24	78.34	35-251	1778044	35.3	32.5	98.87	85.06	0	335.56	255510	99.037	52
9	CFSAN002236	Escherichia coli	SAMN07210945	SRR5805578	862	87.63	16.08	14.09	7.24	78.34	35-251	884930	31.7	29.9	97.6	27.79	0	417.33	77890	98.202	259
9	CFSAN008100	Listeria monocytogenes	SAMN07210931	SRR5805580	862	87.63	16.08	14.09	7.24	78.34	35-251	1202306	35.2	32.6	98.85	69.59	0	439.89	285013	98.6	67
9	CFSAN030807	Shigella sonnei	SAMN07210932	SRR5806400	862	87.63	16.08	14.09	7.24	78.34	35-251	1735668	33.9	30.3	97.81	51.82	0	410.65	20406	88.105	591
9	CFSAN032805	Campylobacter coli	SAMN07210933	SRR5805518	862	87.63	16.08	14.09	7.24	78.34	35-251	1171920	36	33.3	98.83	94.23	0	328.88	82974	98.375	150
9	CFSAN032806	Campylobacter jejuni	SAMN07210930	SRR5805511	862	87.63	16.08	14.09	7.24	78.34	35-251	4300430	36.3	35.7	98.42	148.25	1	150.06	104440	97.194	225
10	CFSAN000255	Salmonella enterica subsp. enterica serovar Montevideo	SAMN07210937	SRR2534386	658	95.88	12.69	12.16	6.25	81.46	35-251	2329734	36.1	34.7	98.53	52.58	1	244.12	440987	99.197	67
10	CFSAN000318	Salmonella enterica subsp. enterica serovar Heidelberg	SAMN07210938	SRR5805862	658	95.88	12.69	12.16	6.25	81.46	35-251	1923960	36	34.9	98.56	40.57	2	238.57	423368	99.144	107
10	CFSAN001178	Listeria monocytogenes	SAMN07210944	SRR2534153	658	95.88	12.69	12.16	6.25	81.46	35-251	2395440	37	36.5	99.23	85.22	2	238.19	476905	99.05	45
10	CFSAN002236	Escherichia coli	SAMN07210945	SRR2534155	658	95.88	12.69	12.16	6.25	81.46	35-251	1062040	36.1	34.2	98.35	21.06	4	228.49	185204	98.241	140
10	CFSAN008100	Listeria monocytogenes	SAMN07210931	SRR2534154	658	95.88	12.69	12.16	6.25	81.46	35-251	1420018	36.9	35.8	98.77	42.24	0	200.88	353282	98.596	59
10	CFSAN030807	Shigella sonnei	SAMN07210932	SRR2534156	658	95.88	12.69	12.16	6.25	81.46	35-251	1716870	36.1	34.3	98.23	30.43	0	219.56	21425	92.467	595
10	CFSAN032805	Campylobacter coli	SAMN07210933	SRR2534157	658	95.88	12.69	12.16	6.25	81.46	35-251	848090	37.2	36.9	99.06	49.81	0	227.51	169981	98.866	76
10	CFSAN032806	Campylobacter jejuni	SAMN07210930	SRR2534158	658	95.88	12.69	12.16	6.25	81.46	35-251	1146398	37.3	37	99.12	64.88	1	236.33	104440	97.216	63
11	CFSAN000255	Salmonella enterica subsp. enterica serovar Montevideo	SAMN07210937	SRR5806452	1307	80.84	23.53	19.06	9.8	73.78	35-251	2524066	34.3	30.1	95.3	51.14	0	264.82	422825	99.16	144
11	CFSAN000255	Salmonella enterica subsp. enterica serovar Montevideo	SAMN07210937	SRR5806454	1307	80.84	23.53	19.06	9.8	73.78	35-251	2176430	34.3	30	95.95	49.38	0	296.04	440987	99.177	120
11	CFSAN000318	Salmonella enterica subsp. enterica serovar Heidelberg	SAMN07210938	SRR5806450	1307	80.84	23.53	19.06	9.8	73.78	35-251	2153018	34.3	30.8	95.88	42.58	0	265.88	248823	99.209	167
11	CFSAN000318	Salmonella enterica subsp. enterica serovar Heidelberg	SAMN07210938	SRR5806434	1307	80.84	23.53	19.06	9.8	73.78	35-251	1945436	34.1	29.5	96.76	51.74	0	354.14	259788	99.225	133
11	CFSAN000318	Salmonella enterica subsp. enterica serovar Heidelberg	SAMN07210938	SRR5806439	1307	80.84	23.53	19.06	9.8	73.78	35-251	2244920	34	30.5	96.15	48.13	0	290.1	259854	99.188	110
11	CFSAN001178	Listeria monocytogenes	SAMN07210944	SRR5805869	1307	80.84	23.53	19.06	9.8	73.78	35-251	2348796	35.9	32.3	98.63	88.23	0	278.99	476905	99.05	86
11	CFSAN001178	Listeria monocytogenes	SAMN07210944	SRR5805867	1307	80.84	23.53	19.06	9.8	73.78	35-251	2683900	35.8	32.7	98.94	107.34	0	290.33	476905	99.05	60
11	CFSAN001178	Listeria monocytogenes	SAMN07210944	SRR5805874	1307	80.84	23.53	19.06	9.8	73.78	35-251	2320362	35.7	32.3	98.91	97.42	0	308.1	476905	99.05	58
11	CFSAN002236	Escherichia coli	SAMN07210945	SRR5805595	1307	80.84	23.53	19.06	9.8	73.78	35-251	2599952	34.9	28.9	94.51	51.41	0	288.78	190130	98.756	147
11	CFSAN002236	Escherichia coli	SAMN07210945	SRR5805594	1307	80.84	23.53	19.06	9.8	73.78	35-251	2210396	34.2	29.1	96.92	57.01	0	349.92	183863	98.755	136
11	CFSAN002236	Escherichia coli	SAMN07210945	SRR5805593	1307	80.84	23.53	19.06	9.8	73.78	35-251	2115694	34.2	30.7	97.36	50.06	0	316.03	183863	98.745	131
11	CFSAN008100	Listeria monocytogenes	SAMN07210931	SRR5805866	1307	80.84	23.53	19.06	9.8	73.78	35-251	2148818	35.8	32.6	98.58	82.33	2	289.09	353282	98.487	115
11	CFSAN008100	Listeria monocytogenes	SAMN07210931	SRR5805877	1307	80.84	23.53	19.06	9.8	73.78	35-251	2418500	35.8	33	98.87	96.43	2	297.87	353282	98.498	98
11	CFSAN030807	Shigella sonnei	SAMN07210932	SRR5806436	1307	80.84	23.53	19.06	9.8	73.78	35-251	2539056	34.8	29.4	95.37	49.29	0	296.96	21397	89.129	543
11	CFSAN030807	Shigella sonnei	SAMN07210932	SRR5806443	1307	80.84	23.53	19.06	9.8	73.78	35-251	1856764	34.3	30.2	96.98	41.74	0	329.56	21397	89.088	522
11	CFSAN030807	Shigella sonnei	SAMN07210932	SRR5806469	1307	80.84	23.53	19.06	9.8	73.78	35-251	2623012	34.5	29.3	94.9	44.06	0	264.55	21425	89.61	511
12	CFSAN000255	Salmonella enterica subsp. enterica serovar Montevideo	SAMN07210937	SRR5806463	920	92.71	17.26	16.01	8.23	82.24	35-251	1365820	34.9	32.2	98.7	50.79	0	417.65	149376	99.03	127
12	CFSAN000255	Salmonella enterica subsp. enterica serovar Montevideo	SAMN07210937	SRR5806470	920	92.71	17.26	16.01	8.23	82.24	35-251	2814482	35.8	33.7	97.99	60.83	0	236.72	440987	99.171	95
12	CFSAN000318	Salmonella enterica subsp. enterica serovar Heidelberg	SAMN07210938	SRR5806466	920	92.71	17.26	16.01	8.23	82.24	35-251	1573848	35.1	32.3	98.51	53.93	0	406.7	114831	99.116	155
12	CFSAN000318	Salmonella enterica subsp. enterica serovar Heidelberg	SAMN07210938	SRR5806460	920	92.71	17.26	16.01	8.23	82.24	35-251	2486336	35.5	34.5	97.87	48.52	0	222.23	259854	99.199	151
12	CFSAN001178	Listeria monocytogenes	SAMN07210944	SRR5805878	920	92.71	17.26	16.01	8.23	82.24	35-251	1296836	36.6	34.4	99.46	74.81	1	398.22	477130	99.054	60
12	CFSAN001178	Listeria monocytogenes	SAMN07210944	SRR5805876	920	92.71	17.26	16.01	8.23	82.24	35-251	2543890	36.7	35.9	99.44	101.47	1	264.88	548130	99.046	68
12	CFSAN002236	Escherichia coli	SAMN07210945	SRR5805592	920	92.71	17.26	16.01	8.23	82.24	35-251	1719000	35.4	33.2	98.95	55.81	0	384.92	148241	98.695	143
12	CFSAN002236	Escherichia coli	SAMN07210945	SRR5805589	920	92.71	17.26	16.01	8.23	82.24	35-251	2549826	35.6	32.9	97.77	53.92	0	252.01	162545	98.728	210
12	CFSAN008100	Listeria monocytogenes	SAMN07210931	SRR5805875	920	92.71	17.26	16.01	8.23	82.24	35-251	1527866	36.6	35.2	99.39	82.6	0	378.53	161120	98.493	116
12	CFSAN008100	Listeria monocytogenes	SAMN07210931	SRR5805879	920	92.71	17.26	16.01	8.23	82.24	35-251	2502086	36.8	35.9	99.23	90.5	0	247.96	353282	98.498	78
12	CFSAN030807	Shigella sonnei	SAMN07210932	SRR5806467	920	92.71	17.26	16.01	8.23	82.24	35-251	1300096	35.1	32.4	98.46	43.65	0	448.18	19757	88.353	557
12	CFSAN030807	Shigella sonnei	SAMN07210932	SRR5806465	920	92.71	17.26	16.01	8.23	82.24	35-251	2413482	35.8	33.1	97.49	46.67	0	253.65	21854	89.06	596
12	CFSAN032805	Campylobacter coli	SAMN07210933	SRR5805522	920	92.71	17.26	16.01	8.23	82.24	35-251	1329598	36.9	35.7	99.53	117.16	0	351.25	77057	98.474	98
12	CFSAN032805	Campylobacter coli	SAMN07210933	SRR5805542	920	92.71	17.26	16.01	8.23	82.24	35-251	2571886	37	35.2	99.13	134.91	0	207.68	130360	98.565	47
12	CFSAN032806	Campylobacter jejuni	SAMN07210930	SRR5805544	920	92.71	17.26	16.01	8.23	82.24	35-251	984576	36.9	35.3	99.22	88.65	1	392.92	62170	96.622	113
12	CFSAN032806	Campylobacter jejuni	SAMN07210930	SRR5805545	920	92.71	17.26	16.01	8.23	82.24	35-251	2432534	36.8	35.8	98.83	106.72	1	186.88	84890	96.764	121
13	CFSAN000255	Salmonella enterica subsp. enterica serovar Montevideo	SAMN07210937	SRR5911617	1260	86.93	23.48	20.41	10.49	80.35	35-251	2076112	34.2	29.4	96.94	74.26	0	433.66	186593	99.117	206
13	CFSAN000255	Salmonella enterica subsp. enterica serovar Montevideo	SAMN07210937	SRR5912121	1260	86.93	23.48	20.41	10.49	80.35	35-251	2952848	34.4	32.2	98.4	112.14	0	434.76	422825	99.141	136
13	CFSAN000318	Salmonella enterica subsp. enterica serovar Heidelberg	SAMN07210938	SRR5942478	1260	86.93	23.48	20.41	10.49	80.35	35-251	3253230	34.5	30.1	97.34	105	0	397.53	231495	99.199	227
13	CFSAN000318	Salmonella enterica subsp. enterica serovar Heidelberg	SAMN07210938	SRR5910642	1260	86.93	23.48	20.41	10.49	80.35	35-251	2773062	34.4	32	98.12	91.06	0	396.12	189616	99.203	150
13	CFSAN001178	Listeria monocytogenes	SAMN07210944	SRR5910650	1260	86.93	23.48	20.41	10.49	80.35	35-251	2219824	35.9	34.1	99.19	118.43	0	374.69	255510	99.031	141
13	CFSAN001178	Listeria monocytogenes	SAMN07210944	SRR5911614	1260	86.93	23.48	20.41	10.49	80.35	35-251	2794420	35.7	33.3	99.16	154.69	0	383.85	477130	99.056	143
13	CFSAN002236	Escherichia coli	SAMN07210945	SRR5910646	1260	86.93	23.48	20.41	10.49	80.35	35-251	3897016	34.8	31.9	98.22	115.52	0	355.99	179966	98.705	196
13	CFSAN002236	Escherichia coli	SAMN07210945	SRR5911616	1260	86.93	23.48	20.41	10.49	80.35	35-251	3104390	34.5	32.9	98.46	102.01	0	401.25	182910	98.71	314
13	CFSAN008100	Listeria monocytogenes	SAMN07210931	SRR5910651	1260	86.93	23.48	20.41	10.49	80.35	35-251	2157266	35.8	33.3	98.98	118.36	0	393.82	353282	98.531	121
13	CFSAN008100	Listeria monocytogenes	SAMN07210931	SRR5912120	1260	86.93	23.48	20.41	10.49	80.35	35-251	2215040	35.8	32.9	98.88	122.72	0	406.05	353282	98.541	142
13	CFSAN030807	Shigella sonnei	SAMN07210932	SRR5910648	1260	86.93	23.48	20.41	10.49	80.35	35-251	3011082	34.8	31.3	97.8	90.14	0	404.43	21306	88.505	598
13	CFSAN030807	Shigella sonnei	SAMN07210932	SRR5947785	1260	86.93	23.48	20.41	10.49	80.35	35-251	2091172	34.4	31.7	97.97	69.1	0	451.6	21765	88.493	673
14	CFSAN000255	Salmonella enterica subsp. enterica serovar Montevideo	SAMN07210937	SRR5805957	877	94.15	16.8	15.82	8.13	82.53	35-251	2284288	35.2	32.3	98.21	69.73	1	343.31	422825	99.169	87
14	CFSAN000255	Salmonella enterica subsp. enterica serovar Montevideo	SAMN07210937	SRR5805960	877	94.15	16.8	15.82	8.13	82.53	35-251	2573584	35.2	29.1	95.76	76.32	1	350.58	227013	99.165	102
14	CFSAN000318	Salmonella enterica subsp. enterica serovar Heidelberg	SAMN07210938	SRR5805605	877	94.15	16.8	15.82	8.13	82.53	35-251	2243876	35.3	32.2	98.16	66.84	0	349.75	213775	99.195	127
14	CFSAN000318	Salmonella enterica subsp. enterica serovar Heidelberg	SAMN07210938	SRR5805604	877	94.15	16.8	15.82	8.13	82.53	35-251	2152998	35.1	31.3	97.76	63.17	0	348.74	203736	99.184	119
14	CFSAN001178	Listeria monocytogenes	SAMN07210944	SRR5805547	877	94.15	16.8	15.82	8.13	82.53	35-251	1974046	36.6	33.4	99.11	102.14	0	355.18	477130	99.061	56
14	CFSAN001178	Listeria monocytogenes	SAMN07210944	SRR5805549	877	94.15	16.8	15.82	8.13	82.53	35-251	1907038	36.5	33.5	99.2	100.58	0	360.86	548130	99.046	54
14	CFSAN002236	Escherichia coli	SAMN07210945	SRR5805551	877	94.15	16.8	15.82	8.13	82.53	35-251	2297310	35.1	33.2	98.61	70.11	0	362.07	183863	98.684	139
14	CFSAN002236	Escherichia coli	SAMN07210945	SRR5805554	877	94.15	16.8	15.82	8.13	82.53	35-251	2243944	35.5	32.7	98.39	63.37	0	339.98	180419	98.753	135
14	CFSAN008100	Listeria monocytogenes	SAMN07210931	SRR5805553	877	94.15	16.8	15.82	8.13	82.53	35-251	1711478	36.4	35	99.28	89.25	0	367.72	353282	98.622	75
14	CFSAN008100	Listeria monocytogenes	SAMN07210931	SRR5805557	877	94.15	16.8	15.82	8.13	82.53	35-251	1177062	36.2	32.7	98.77	61.41	0	374.02	161124	98.461	670
14	CFSAN030807	Shigella sonnei	SAMN07210932	SRR5805959	877	94.15	16.8	15.82	8.13	82.53	35-251	1103458	35.1	32.5	98.03	30.41	0	388.23	19230	88.336	566
14	CFSAN030807	Shigella sonnei	SAMN07210932	SRR5805955	877	94.15	16.8	15.82	8.13	82.53	35-251	2337484	35.6	32.4	98.18	59.47	0	329.47	21904	88.773	526
14	CFSAN032805	Campylobacter coli	SAMN07210933	SRR5805479	877	94.15	16.8	15.82	8.13	82.53	35-251	1847850	36.8	34.3	99.28	134.85	0	289.99	165134	98.791	101
14	CFSAN032805	Campylobacter coli	SAMN07210933	SRR5805459	877	94.15	16.8	15.82	8.13	82.53	35-251	2283742	37.1	35.1	99.3	149.42	0	259.73	169981	98.921	73
14	CFSAN032806	Campylobacter jejuni	SAMN07210930	SRR5805463	877	94.15	16.8	15.82	8.13	82.53	35-251	909816	36.8	34.1	98.76	62.99	2	290	51498	96.897	1315
14	CFSAN032806	Campylobacter jejuni	SAMN07210930	SRR5805460	877	94.15	16.8	15.82	8.13	82.53	35-251	1220878	35.8	34.1	99.12	85.02	2	292.2	104440	97.226	76
15	CFSAN000255	Salmonella enterica subsp. enterica serovar Montevideo	SAMN07210937	SRR5806412	NA	NA	NA	NA	NA	NA	35-251	3243820	34.7	32.1	98.02	94.05	0	314.54	440987	99.18	107

15	CFSAN000318	Salmonella enterica subsp. enterica serovar Heidelberg	SAMN07210938	SRR5806418	NA	NA	NA	NA	NA	NA	35-251	1918662	34.3	32.6	98.53	61.37	0	363.05	259854	99.218	121
15	CFSAN001178	Listeria monocytogenes	SAMN07210944	SRR5805852	414	96.41	7.98	7.69	3.95	84.75	35-251	794634	37.1	35.2	99.36	46.85	0	454.23	272633	99.039	49
15	CFSAN002236	Escherichia coli	SAMN07210945	SRR5805585	414	96.41	7.98	7.69	3.95	84.75	35-251	1090220	36.3	33.4	98.73	36.86	0	471.59	161548	98.703	129
15	CFSAN008100	Listeria monocytogenes	SAMN07210931	SRR5805851	414	96.41	7.98	7.69	3.95	84.75	35-251	633854	36.9	33.5	98.69	36.81	0	471.67	285013	98.473	251
15	CFSAN030807	Shigella sonnei	SAMN07210932	SRR5806415	414	96.41	7.98	7.69	3.95	84.75	35-251	907740	36.2	33.2	98.54	31.69	0	524.37	21828	88.676	538
15	CFSAN032805	Campylobacter coli	SAMN07210933	SRR5805543	414	96.41	7.98	7.69	3.95	84.75	35-251	619518	37.4	35.4	99.42	59.44	0	420.74	169809	98.769	57
15	CFSAN032806	Campylobacter jejuni	SAMN07210930	SRR5805541	414	96.41	7.98	7.69	3.95	84.75	35-251	635066	37.3	36.1	62.03	31.84	1	362.46	45937	96.686	485
16	CFSAN000255	Salmonella enterica subsp. enterica serovar Montevideo	SAMN07210937	SRR5806401	1279	85.32	24.38	20.8	10.69	72.12	35-251	2486162	33.6	30.1	97.74	82.2	0	382.92	364987	99.171	104
16	CFSAN000318	Salmonella enterica subsp. enterica serovar Heidelberg	SAMN07210938	SRR5805854	1279	85.32	24.38	20.8	10.69	72.12	35-251	2409148	33.3	29.3	97.19	72.39	1	376.53	423434	99.218	137
16	CFSAN001178	Listeria monocytogenes	SAMN07210944	SRR5805860	1279	85.32	24.38	20.8	10.69	72.12	35-251	1854464	34.9	32	99.14	93.87	0	347.72	476905	99.03	90
16	CFSAN002236	Escherichia coli	SAMN07210945	SRR5805586	1279	85.32	24.38	20.8	10.69	72.12	35-251	3234242	33.6	29.1	96.71	82.73	2	331.54	185204	98.751	151
16	CFSAN008100	Listeria monocytogenes	SAMN07210931	SRR5805850	1279	85.32	24.38	20.8	10.69	72.12	35-251	1830302	34.7	30.7	98.54	81.51	0	324.53	353282	98.507	122
16	CFSAN030807	Shigella sonnei	SAMN07210932	SRR5806410	1279	85.32	24.38	20.8	10.69	72.12	35-251	3467008	33.7	28.5	95.22	71.66	0	323.46	21862	89.122	568
16	CFSAN032805	Campylobacter coli	SAMN07210933	SRR5805519	1279	85.32	24.38	20.8	10.69	72.12	35-251	1729202	35.6	32.7	98.89	114.95	0	266.79	165134	98.748	138
16	CFSAN032806	Campylobacter jejuni	SAMN07210930	SRR5805520	1279	85.32	24.38	20.8	10.69	72.12	35-251	1147182	35.4	30.9	98.46	87.07	1	334.01	64862	97.075	150
17	CFSAN000255	Salmonella enterica subsp. enterica serovar Montevideo	SAMN07210937	SRR5806429	1220	91.27	23.12	21.1	10.85	80.52	35-251	1929840	35.3	33.3	97.96	43.17	0	243.26	440987	99.181	135
17	CFSAN000255	Salmonella enterica subsp. enterica serovar Montevideo	SAMN07210937	SRR5806426	1220	91.27	23.12	21.1	10.85	80.52	35-251	3539004	35.4	33.1	98.03	83.38	0	259.17	440987	99.184	143
17	CFSAN000318	Salmonella enterica subsp. enterica serovar Heidelberg	SAMN07210938	SRR5806427	1220	91.27	23.12	21.1	10.85	80.52	35-251	1328786	34.7	29.7	96.97	45.94	0	436.62	259788	99.214	150
17	CFSAN000318	Salmonella enterica subsp. enterica serovar Heidelberg	SAMN07210938	SRR5806422	1220	91.27	23.12	21.1	10.85	80.52	35-251	2575330	35.1	32.8	98.27	69.3	0	314.8	259743	99.231	154
17	CFSAN001178	Listeria monocytogenes	SAMN07210944	SRR5805865	1220	91.27	23.12	21.1	10.85	80.52	35-251	2074390	36.4	34.4	99.45	112.51	1	373.47	548130	99.046	73
17	CFSAN001178	Listeria monocytogenes	SAMN07210944	SRR5805871	1220	91.27	23.12	21.1	10.85	80.52	35-251	2749682	36.4	34.7	99.45	127.16	1	312.9	477130	99.056	61
17	CFSAN002236	Escherichia coli	SAMN07210945	SRR5805576	1220	91.27	23.12	21.1	10.85	80.52	35-251	1569012	35.5	34	98.34	34.47	1	256.77	190130	98.778	121
17	CFSAN002236	Escherichia coli	SAMN07210945	SRR5805590	1220	91.27	23.12	21.1	10.85	80.52	35-251	2246446	35.5	33.8	98.37	50.4	2	263.81	190130	98.799	124
17	CFSAN008100	Listeria monocytogenes	SAMN07210931	SRR5805868	1220	91.27	23.12	21.1	10.85	80.52	35-251	3710430	36.4	35.1	99.23	166.4	0	315.13	355825	98.413	209
17	CFSAN008100	Listeria monocytogenes	SAMN07210931	SRR5805872	1220	91.27	23.12	21.1	10.85	80.52	35-251	1832204	36.1	33.1	98.93	110.44	0	453.49	348077	98.524	177
17	CFSAN030807	Shigella sonnei	SAMN07210932	SRR5806424	1220	91.27	23.12	21.1	10.85	80.52	35-251	3127086	35.7	33.6	97.92	58.42	0	242.29	21397	89.281	511
17	CFSAN030807	Shigella sonnei	SAMN07210932	SRR5806437	1220	91.27	23.12	21.1	10.85	80.52	35-251	2604576	35.6	34.2	98.28	54.55	1	267.44	22029	89.314	519
18	CFSAN000318	Salmonella enterica subsp. enterica serovar Heidelberg	SAMN07210938	SRR5805847	986	91.49	NA	NA	NA	83.5	35-251	3056870	35	32.3	98.17	88.22	0	342.79	266303	99.201	127
18	CFSAN001178	Listeria monocytogenes	SAMN07210944	SRR5805572	986	91.49	NA	NA	NA	83.5	35-251	2063854	36.5	34.1	99.14	101.32	3	338.35	255509	99.053	68
18	CFSAN002236	Escherichia coli	SAMN07210945	SRR5805568	986	91.49	NA	NA	NA	83.5	35-251	2165412	34.7	31.5	98.36	69.42	0	401.75	176340	98.722	166
18	CFSAN008100	Listeria monocytogenes	SAMN07210931	SRR5805567	986	91.49	NA	NA	NA	83.5	35-251	2089152	35.7	33.1	99.05	97.63	1	328.31	353282	98.626	90
18	CFSAN030807	Shigella sonnei	SAMN07210932	SRR5805967	986	91.49	NA	NA	NA	83.5	35-251	2377832	35.1	32.7	98.21	66.02	0	365.34	21898	88.743	547
18	CFSAN032805	Campylobacter coli	SAMN07210933	SRR5805478	986	91.49	NA	NA	NA	83.5	35-251	1747534	36.8	36	99.45	133.91	0	302.39	86641	98.764	226
18	CFSAN032806	Campylobacter jejuni	SAMN07210930	SRR5805462	986	91.49	NA	NA	NA	83.5	35-251	2462464	36.9	36.1	99.23	135.55	1	242.19	51766	96.479	102
19	CFSAN000255	Salmonella enterica subsp. enterica serovar Montevideo	SAMN07210937	SRR5805963	918	91.19	NA	NA	8.1	82.1	35-251	3322360	34.9	31.1	98.05	130.77	1	476.05	684920	99.196	92
19	CFSAN000318	Salmonella enterica subsp. enterica serovar Heidelberg	SAMN07210938	SRR5805846	918	91.19	NA	NA	8.1	82.1	35-251	2352160	34.8	29.4	96.82	89.18	0	511.14	437894	99.216	105
19	CFSAN001178	Listeria monocytogenes	SAMN07210944	SRR5805560	918	91.19	NA	NA	8.1	82.1	35-251	1079018	36.3	33.3	99.25	70.68	3	485.04	548130	99.057	87
19	CFSAN002236	Escherichia coli	SAMN07210945	SRR5805556	918	91.19	NA	NA	8.1	82.1	35-251	2661454	34.9	32.6	98.79	104.46	0	518.81	189159	98.743	249
19	CFSAN008100	Listeria monocytogenes	SAMN07210931	SRR5805559	918	91.19	NA	NA	8.1	82.1	35-251	790864	36.2	31.3	98.33	46.99	0	441.66	189826	98.547	120
19	CFSAN030807	Shigella sonnei	SAMN07210932	SRR5805964	918	91.19	NA	NA	8.1	82.1	35-251	993890	34.7	31.1	97.51	33.96	0	539.11	21674	88.394	607
19	CFSAN032805	Campylobacter coli	SAMN07210933	SRR5805480	918	91.19	NA	NA	8.1	82.1	35-251	754330	36.7	35.6	99.52	81.69	1	449.57	165134	98.717	211
19	CFSAN032806	Campylobacter jejuni	SAMN07210930	SRR5805481	918	91.19	NA	NA	8.1	82.1	35-251	697948	36.6	34.7	99.28	61.62	1	380.69	62739	96.944	195
20	CFSAN000255	Salmonella enterica subsp. enterica serovar Montevideo	SAMN07210937	SRR5805970	828	92.62	15.7	14.54	7.44	80.47	35-250	1698042	34.8	32.3	98.47	63.75	0	481.38	511299	99.179	67
20	CFSAN000318	Salmonella enterica subsp. enterica serovar Heidelberg	SAMN07210938	SRR5805849	828	92.62	15.7	14.54	7.44	80.47	35-250	3067102	35.4	33.5	98.47	90.59	0	363.57	725244	99.218	115
20	CFSAN001178	Listeria monocytogenes	SAMN07210944	SRR5805565	828	92.62	15.7	14.54	7.44	80.47	35-250	1376904	36.7	35.2	99.01	54.05	0	269.54	548130	99.042	71
20	CFSAN002236	Escherichia coli	SAMN07210945	SRR5805575	828	92.62	15.7	14.54	7.44	80.47	35-250	2841996	35.5	34.6	98.57	70.13	0	297.04	186967	98.79	163
20	CFSAN008100	Listeria monocytogenes	SAMN07210931	SRR5805573	828	92.62	15.7	14.54	7.44	80.47	35-250	1483720	36.4	35.6	99.2	68.61	0	330.94	353282	98.519	116
20	CFSAN030807	Shigella sonnei	SAMN07210932	SRR5805966	828	92.62	15.7	14.54	7.44	80.47	35-250	2291552	35.6	34.5	98.21	52.98	0	307.21	21425	88.865	522
20	CFSAN032805	Campylobacter coli	SAMN07210933	SRR5805515	828	92.62	15.7	14.54	7.44	80.47	35-250	397922	36.4	33	99.01	41.27	0	448.21	216026	98.87	65
20	CFSAN032806	Campylobacter jejuni	SAMN07210930	SRR5805513	828	92.62	15.7	14.54	7.44	80.47	35-250	693996	36.8	34.5	99.05	52.42	1	326	104440	97.246	135
21	CFSAN000255	Salmonella enterica subsp. enterica serovar Montevideo	SAMN07210937	SRR5805968	896	91.85	NA	NA	NA	80.96	35-251	2188002	35	30.4	98.08	85.07	0	480.48	422657	99.163	74
21	CFSAN000318	Salmonella enterica subsp. enterica serovar Heidelberg	SAMN07210938	SRR5805845	896	91.85	NA	NA	NA	80.96	35-251	2138130	35.1	30.5	97.89	76.67	0	476.5	259788	99.196	120
21	CFSAN001178	Listeria monocytogenes	SAMN07210944	SRR5805566	896	91.85	NA	NA	NA	80.96	35-251	1979908	36.4	31.4	98.6	116.92	1	445.93	265665	99.019	61
21	CFSAN002236	Escherichia coli	SAMN07210945	SRR5805570	896	91.85	NA	NA	NA	80.96	35-251	1925110	35.1	29.7	97.74	68.99	0	499.06	158690	98.697	156
21	CFSAN008100	Listeria monocytogenes	SAMN07210931	SRR5805569	896	91.85	NA	NA	NA	80.96	35-251	1728250	36.2	33.9	99.25	101.99	0	453.98	285013	98.573	76
21	CFSAN030807	Shigella sonnei	SAMN07210932	SRR5805965	896	91.85	NA	NA	NA	80.96	35-251	2740768	35.5	33.6	98.36	79.76	0	404.65	21674	88.534	526
21	CFSAN032805	Campylobacter coli	SAMN07210933	SRR5805461	896	91.85	NA	NA	NA	80.96	35-251	1549158	36.7	35.5	99.49	140.19	0	389.62	106055	98.844	127
21	CFSAN032806	Campylobacter jejuni	SAMN07210930	SRR5805516	896	91.85	NA	NA	NA	80.96	35-251	1706794	36.6	35.4	99.34	135.01	1	353.39	73169	96.81	49

Supplemental Table 2. Run metrics submitted by the labs.

Lab	ClusterDensity	PercClustersPF	Reads_M	ReadsPF_M	Yield	Q30
1	463	97.46	9.04	8.81	4.6	91.3
2	NA	NA	NA	NA	NA	NA
3	NA	NA	NA	NA	NA	NA
4	887	90.84	16.99	15.44	7.93	84.15
5	909	91.22	17.02	15.52	7.98	83.53
6	1203	88.51	22.69	20.09	10.32	75.57
6	1335	88.97	25.19	22.42	11.52	73.12
11	1307	80.84	23.53	19.06	9.8	73.78
9	862	87.63	16.08	14.09	7.24	78.34
10	658	95.88	12.69	12.16	6.25	81.46
13	1260	86.93	23.48	20.41	10.49	80.35
12	920	92.71	17.26	16.01	8.23	82.24
8	NA	NA	NA	NA	NA	NA
7	927	94.53	17.71	16.74	8.6	86.8
17	1220	91.27	23.12	21.1	10.85	80.52
14	877	94.15	16.8	15.82	8.13	82.53
15	414	96.41	7.98	7.69	3.95	84.75
16	1279	85.32	24.38	20.8	10.69	72.12
20	828	92.62	15.7	14.54	7.44	80.47
18	986	91.49	NA	NA	NA	83.5
19	918	91.19	NA	NA	8.1	82.1
21	896	91.85	NA	NA	NA	80.96
**AVG:**	**955**	**91**	**18**	**16**	**8**	**81**

Supplemental Table 3. SNP positions with respect to reference genome.

Reference genome	Replicon	SNP position	Number of PT submissions with SNP
CFSAN000255	chromosome	2636095	2
CFSAN000255	chromosome	2636324	3
CFSAN000255	chromosome	2636432	1
CFSAN000318	chromosome	1163678	1
CFSAN000318	chromosome	1522567	1
CFSAN000318	chromosome	3844854	1
CFSAN000318	chromosome	4213567	5
CFSAN001178	chromosome	71247	1
CFSAN001178	chromosome	203018	1
CFSAN001178	chromosome	908902	1
CFSAN001178	chromosome	1530728	2
CFSAN001178	chromosome	1676551	2
CFSAN001178	chromosome	1677026	1
CFSAN001178	chromosome	1903451	1
CFSAN001178	chromosome	2562265	1
CFSAN001178	chromosome	2704336	9
CFSAN001178	chromosome	2720398	1
CFSAN001178	chromosome	2756164	1
CFSAN002236	chromosome	758652	1
CFSAN002236	chromosome	804517	2
CFSAN002236	chromosome	1839592	2
CFSAN002236	chromosome	3690530	1
CFSAN002236	chromosome	3697978	1
CFSAN002236	plasmid_pO104_H21	156546	6
CFSAN008100	chromosome	187043	2
CFSAN008100	chromosome	1397275	2
CFSAN008100	chromosome	1955083	1
CFSAN030807	chromosome	1932106	1
CFSAN032805	chromosome	422302	1
CFSAN032806	chromosome	634014	18
CFSAN032806	chromosome	775060	2

Supplemental Table 4. Spearman correlation table of run metrics and summary statistics. Significant positive and negative correlations are represented as + and -, respectively. The size of the symbols and font boldness represent the degree of correlation.

Spearman

	Reads	MeanForRQ	MeanRevRQ	ReadsMapped(%)	MeanDepth	SNPs	MeanInsert	NG50	GenomeFraction	Contigs	ClusterDensity	ClustersPF(%)	Reads(Mil)	ReadsPF(Mil)	ReadsPF(%)	Yield	Q30
**Reads**		-	-	-	+		-	+	+		+	-	+	+	-	+	-
**MeanForRQ**	-		+	+	+	+	-		-	-	-	+	-	-	+	-	+
**MeanRevRQ**	-	+		+	+	+	-		-	-	-	+	-	-	+	-	+
**ReadsMapped(%)**	-	+	+		+	+				-	-	+	-	-	+	-	+
**MeanDepth**	+	+	+	+				+		-	+	-	+	+	-	+	
**SNPs**		+	+	+						-							
**MeanInsert**	-	-	-								-			-		-	+
**NG50**	+				+				+	-	+		+	+		+	
**GenomeFraction**	+	-	-					+		-							
**Contigs**		-	-	-	-	-		-	-								
**ClusterDensity**	+	-	-	-	+		-	+				-	+	+	-	+	-
**ClustersPF(%)**	-	+	+	+	-						-		-	-	+	-	+
**Reads(Mil)**	+	-	-	-	+			+			+	-		+	-	+	-
**ReadsPF(Mil)**	+	-	-	-	+		-	+			+	-	+		-	+	-
**ReadsPF(%)**	-	+	+	+	-						-	+	-	-		-	+
**Yield**	+	-	-	-	+		-	+			+	-	+	+	-		-
**Q30**	-	+	+	+			+				-	+	-	-	+	-	

rho <0.5	+
0.5 >= rho < 0.75	+
rho >= 0.75	+
